# Management of chronic non-healing venous stasis ulcer through Siddha intervention - a case report

**DOI:** 10.1016/j.jaim.2024.101034

**Published:** 2024-12-04

**Authors:** Nikil Niva John Raja, Hema Nandhini Devi Veluchamy, Muthu Vignesh Sivanan

**Affiliations:** Siddha Medical Consultant, Muthu Siddha Hospital, Seranmahadevi, Tirunelveli, Tamilnadu, India

**Keywords:** Case report, Siddha, Venous ulcer, *Parangipattai chooranam*, *Maththan thylam*

## Abstract

Venous stasis ulcer is a chronic vascular disease and majorly occurred in lower limbs. Recurrence of ulcer in lower extremities are common in venous stasis ulcer. A 65 years old male patient had a symptoms of non-healing ulcer in right leg nearby right ankle joint in more than three months and associated with itching, hyperpigmentation and pain. A patient had a previous history of Deep Vein Thrombosis. Significant healing in wound observed under the siddha medication such as combination of Siddha Sastric chooranam (*Parangipattai chooranam, Palagarai parpam, Irunelli Karpam, Sangu parpam*) and *Vallarai* tablet for internal medication and *Triphala chooranam* as external medication for washing wounds whereas, *Maththan thylam* is applied for wound dressing. No recurrence of ulcer was observed after a follow-up of 4 months period. This case report depicted the management of chronic ulcer through Siddha medicines.

## 1 Introduction

Venous Stasis Ulcer (VSU) is a chronic lower extremity disorder caused by chronic venous insufficiency (CVI) due to venous hypertension [1]. Chronic invert venous blood circulation or inhibit venous blood circulation leads to venous hypertension [2]. Venous stasis ulcer is majorly occurred in between the knee to ankle region. Old age peoples, sex, occupation, obesity, trauma, immobility, Deep vein thrombosis (DVT), phlebitis are the risk factors for the development of venous Stasis Ulcer [3]. The patients who suffer from VSU, they have pain, itching, aching, swelling, hyperpigmentation, dermal fibrosis clinical features [4].

Data from Australia, in yearly more than AUD$ 3 billion costs spend in Venous Leg Ulcer (VLU) treatments and £941 million in UK [5]. While there are few Indian studies on the epidemiology of chronic wounds, one study estimated the prevalence at 4.5/1000 population. The incidence of acute wounds was more than double at 10.5/1000 population [6]. In conventional systems, supportive care such as wound dressing, elastic bandages, and compression stocking, self-care includes leg elevation, and physical exercise is being used apart from medications like antibiotics, dietary supplements, and non-steroidal anti-inflammatory drugs. Surgical treatment includes skin grafting, sclerotherapy, Laser ablation, correction of the venous plexus through reconstructive surgery is practiced which have their limitations [7]. In the Siddha system of medicine, World Health Organization Siddha Standard Terminologies describes Venous Stasis Ulcers are correlated with *Natipunn* and Chronic Non- Healing ulcer is correlated with *Aaraapun*. *Aaraapun* is also called as *Pohaa viranam, Pandai viranam, Azhiyatha viranam* [8]. Many Siddha Sastric preparations are described in Siddha literature for Chronic non-healing Venous ulcers. Herein the details of chronic non healing venous stasis ulcer case have been presented, which successively intervened with siddha Sastric medications. This case study observed, ulcer is completely healed in after 8 weeks of siddha intervention and other clinical features severe itching, hyperpigmentation, pain, swelling is completely recovered. Present case report showed Siddha interventions are significant wound healing process in present case and reduce the cost effective for the management of Chronic non-healing Venous stasis ulcer.

## 2 Patient information

A 65-year-old, non-diabetic, non-hypertensive male with no other comorbidities and autoimmune disorders, working as supervisor in construction department with average standing posture for 6–7 hours per day, reported on June 18, 2022 with the complaints of nonhealing ulcer present in the medial compartment of right leg nearby right ankle joint and associated with the complaints of severe itching, hyperpigmentation, pain in right leg and swelling present in right knee joint to right ankle joint in the past 3 months from Muthu siddha hospital Seranmahadevi, Tirunelveli District, Tamil Nadu. Patient had no history of alcohol consumption and smoking habits. Before 2 years patient had mild itching and pain in posterior compartment of right knee joint. Some days after pain and itching aggravated and hyperpigmentation patches present in medial compartment of right leg, then patient visit near allopathic hospital and doctor prescribed medications. But symptoms are worsened in due course with ulceration. He had a consultation with Allopathy doctor and advised to take Doppler study in Lower limb Right leg (venous and artery). It was diagnosed by Deep Vein Thrombosis (DVT) with the help of Doppler report and clinical manifestations. After 4 months allopathic medications ulceration and other clinical findings are recovered. After 2 years patient had same clinical features with ulceration present in right leg. Due to vain experienced by the patient he came to Muthu Siddha Hospital for alternative care.

## 3 Clinical findings

The patient had nonhealing ulcer present in the medial compartment of right leg nearby right ankle joint and associated with the complaints of severe itching, hyperpigmentation, pain in right leg and pain aggravated while walking and swelling present in right knee joint to right ankle joint in the last 3 months. On examination, dilated veins are present in posterior compartment of right leg near right knee joint. Patient vitals were within normal limits.

During general and systemic examinations, Cardio respiratory, Musculoskeletal, Neurological and genitourinary system examinations were normal. On Skin examination ulceration, mild oozing, hyperpigmentation and mild tenderness noted in right ankle joint.

## 4 Timeline

Table 1 represents the timeline of the occurrence of events in the present case study. It represents all the symptoms along with the previous treatment taken by the patient and the results obtained.

**Table 1:** Timeline

Year	Incidence/Intervention
2020	Itching, hyperpigmentation and ulceration present in right leg, recovered after allopathic treatment.
2020–2022	Occasionally pain and mild itching present in right leg. Consulted nearby hospital and take allopathic medication.
June 18, 2022	The patient came to Muthu Siddha Hospital with the complaints of non-healing ulcer present in right leg and hyperpigmentation, itching and pain and swelling in right leg and constipation. Patient was treated with following Siddha Internal and External Medicines: **Internal Medicines**-1. combination of chooranam: *Parangipattai chooranam* (100 gm), *Palagarai parpam* (10 gm), *Sangu parpam* (10 gm), *Irunelli Karpam* (10 gm). Combination chooranam 1 gm twice a day after a meal with milk. 2. *Vallarai* Tablet-2 tablets (500 mg each) twice after a meal with water. **External Medicines**- *Thripala Chooranam* was used for external wash twice a day. Patient was advised to add 5 g of *Thripala Chooranam* to 250 ml of water, boiled it for 10 minutes. Then the ulcer area washed in mild heat and cleaned completely. After *Thripala Chooranam wash Maththan Thailam* was applied to over the affected area.

## 5 Diagnostic findings

Based on the clinical features, clinical assessments, signs and investigations, previous history of venous ulceration the patient diagnosis was confirmed to chronic non-healing venous ulcer.

After examination, as per Siddha aspect the patient was diagnosed as *Natipunn.* As per Siddha literature the clinical features of *Natipunn* are *Narambugalil silaiyodal* (dilated veins), *Aaraa pun (chronic ulcer), Namaichal* (Itching), *Karumpulli (*Hyperpigmentation), *Vali* (Pain), *Kuruthiyum seezhum Vadithal* (pus and bleeding discharge) and *Enbu varai silaiyodal* (Deep wound formation).

Clinical features of the patient correlate with the clinical features of *Natipunn* such as *Aaraa pun (chronic ulcer), Narambugalil silaiyodal* (dilated veins), *Namaichal* (Itching), *Karumpulli (*Hyperpigmentation), *Vali* (Pain). As per Siddha consultation, the patient was diagnosed with Eight-fold diagnostic methods like Naadi- Piththakabam, Naa- Normal, Niram- Normal, Sparisam-affected (Ulceration present in right leg), Mozhi- Normal no abnormal sound, Vizhi- Normal, Malam- Affected (Constipation), Moothiram- Normal.

**Clinical assessment:** Itching, Hyperpigmentation, Pain, Pus Discharge and Swelling was done in before and after treatment. The method of clinical assessment is represented in Table 2, Table 5.

**Table 2:** Method of clinical assessment.

Parameters	Grade			
	0	1	2	3
Pain (VAS scale)	No Pain	Mild	Moderate	Severe
Hyperpigmentation	Absent	Mild	Moderate	Severe
Itching	Absent	Mild	Moderate	Severe
Pus Discharge	Absent	Mild	Moderate	Severe
Swelling	Absent	Mild	Moderate	Severe

S**igns and investigations**:

The severity of the ulcer was measured by the Diabetic Ulcer Severity Score (Ref. Table 3). Classification of Ulcer was measured by the Wagner Ulcer Classification System [9]. It represents Table 4. Characteristics of ulcer and patient satisfaction were measured by the ‘Leg Ulcer Measurement Tool [10]. Blood Investigations: CBC, ESR, HB, Liver Function Test, Renal Function Test, Lipid Profile and Urine Routine Examination. It represents Table 6.

**Table 3:** Diabetic ulcer severity score.

Parameter	Score 0	Score 1
Palpable pedal pulses	Presence	Absence
Probing to bone	No	Yes
Ulcer Site	Toes	Foot
Ulcer Number	Single	Multiple

**Table 4:** Wagner ulcer classification system.

Wagner Ulcer Classification	
Grade 0	Skin intact but bony deformities lead to “foot at risk”
Grade 1	Superficial ulcer
Grade 2	Deeper, full thickness extension
Grade 3	Deep abscess formation or osteomyelitis
Grade 4	Partial gangrene of forefoot
Grade 5	Extensive Gangrene

**Table 5:** Clinical Assessment before treatment and after treatment (after 60 days).

Clinical Assessment	Before treatment Day 0 (1st visit)	Day 15 (1st follow-up visit)	Day 30 (2nd follow-up visit)	Day 45 (3rd follow-up visit)	Day 60 (4th follow-up visit)
Pain (VAS scale)	2	1	1	0	0
Hyperpigmentation	2	2	1	0	0
Itching	3	2	1	0	0
Pus Discharge	1	0	0	0	0
Swelling	1	1	0	0	0

**Table 6:** Investigations before treatment and after treatment.

Investigations	Before Treatment	After Treatment (after 60 days)
**Blood Investigations**		
Neutrophils	56%	59%
Lymphocytes	28%	26%
Eosinophils	8%	02%
Monocytes	06%	03%
Basophils	00%	00%
Haemoglobin	14 g/dL	16 g/dL
RBC Count	4.70 millions/cu. mm	5.15 millions/cu. mm
Platelet count	3.15 lacs cells/cmm	3.75 lacs cells/cmm
ESR	31 mm/hr	14 mm/hr
CRP	15 Mg/l	6 Mg/l
Total WBC Count	7900 cells/cu.mm	8300 cells/cu.mm
Random Blood Sugar	112 mg/dl	104 mg/dl
T. Bilirubin	0.8 mg/dl	0.7 mg/dl
D. Bilirubin	0.2 mg/dl	01. mg/dl
In. Bilirubin	0.6 mg/dl	0.6 mg/dl
SGOT	27 U/L	23 U/L
SGPT	37 U/L	34 U/L
ALP	91 U/L	82 U/L
T. Protein	7.35 gm/dl	7.21 gm/dl
Globulin	2.15 gm/dl	2.6 gm/dl
Albumin	4.52 gm/dl	4.32 gm/dl
T. Cholesterol	210 mg/dL	165 mg/dL
Triglycerides	90 mg/dL	78 mg/dL
Serum Urea	14 mg/dL	13 mg/dL
Serum Creatinine	0.7 mg/dL	0.7 mg/dL
Serum Uric Acid	4.2 mg/dL	3.8 mg/dL
**Urine Routine Analysis**		
Appearance	Clear	Clear
Colour	Pale yellow	Pale yellow
pH	5.3	5.3
Specific Gravity	1.010	1.009
Glucose	Nil	Nil
Bile Salt	Absent	Absent
Bile Pigments	Absent	Absent
Urobilinogen	Normal	Normal
Protein	Nil	Nil
**Urine Microscopic Examination**		
Crystals	Absent	Absent
RBC’S/Hpf	Absent	Absent
Pus Cells/Hpf	1-2/hpf	1-2/Hpf

## 6 Treatment schedule

According to the Siddha Standard Treatment Guidelines published by National Institute of Siddha, ministry of Ayush, Government of India, both Internal and External medicines are recommended for the line of treatment for chronic non-healing ulcer. Ref. Table 7 [11].

**Table 7:** Internal and External medicines are recommended for the line of treatment for chronic non-healing ulceraccording to the Siddha Standard Treatment Guidelines.

Internal Medicines	
S. No	Name of the Medicine/Dose/Adjuvant/Time
1.	***A. Chooranam:***
	*1. Parangipattai Chooranam -* 1–2 gm with warm milk, BD, after food
	*2.Elathy Chooranam-* 1–2 gm with warm milk, BD, after food
	*3. Seenthil Chooranam-* 1–2 gm with warm milk, BD, after food
	*4. Amukkara Chooranam-* 1–2 gm with lukewarm water, BD, after food
	*5. Vallarai Chooranam-* 1–2 gm with lukewarm water, BD, after food.
	*6. Thiripala Chooranam-* 1–2 gm with honey, BD, after food.
	*7. Nilavagai Chooranam (*1–2 gm with lukewarm water, BD, after food)
	***B. Parpam:***
	*1.Palagarai parpam -* 200–400 mg with ghee/warm milk, BD, after food
	*2. sangu parpam-* 200–400 mg with ghee/warm milk, BD, after food.
	***C. Chendooram:***
	*1. Kalameganarayana chendooram* - 30–130 mg, BD, after food.
	***D. Mezhugu:***
	***1.****Gandhi mezhugu* - 200–500 mg with milk, BD, after food
	2. *Rasagandhi mezhugu* - 250–500 mg with palm jaggery, BD, after food.
	***E. Rasayanam:***
	**1.** Gandhaga Rasayanam- 1–3 gm with palm jaggery/ghee, BD, after food
	*2. Parangipattai rasayanam*- 3–6 gm, BD, after food**.**
	***F. Karuppu****:*
	*1. Sivanar amirtham* - 100–200 mg with honey, BD, after food
	2. *Irunelli Karpam*- 100–200 mg with honey, BD, after food**.**
	**Thailam *(Medicated Internal oils):****Rasa thailam, Karappan thailam, Meganatha Thailam.*
**External Medicines**	
1.	***A. Thailam (Medicated External oils):***
	*1. Viranasanjeevi thailam*
	*2. Voonpoochu thailam*
	*3.Maththan Thailam*
	*4. Arugan Thailam*.
	**B. External wash**:
	1. *Thripala Chooranam*
	*2. Padigara Nheer*

The following Siddha sastric medicines were selected for this case report in accordance with the guidelines prescribed by the Siddha medicines for *Nattipun and Aaraapun*.

A combination of chooranam is a unique siddha Sastric medicines which includes *Parangipattai chooranam* (100 gm), *Palagarai parpam* (10 gm), *Sangu parpam* (10 gm), *Irunelli Karpam* (10 gm). Combination chooranam 1 gm twice a day after a meal with milk. *Vallarai* Tablet 2 tablets (500 mg each) twice after a meal with water. *Thripala Chooranam* was used for external wash twice a day. Patient was advised to add 5 g of *Thripala Chooranam* to 250 ml of water, boiled it for 10 minutes. Then the ulcer area washed in mild heat and cleaned completely. After *Thripala Chooranam wash Maththan Thailam* was applied to over the affected area.

Patient was advised to visit the hospital once in 15 days and during each visit, clinical assessments and prognosis was noted. The patient was advised to follow *Pathiyam* in this line of treatment. In Pathiyam patient advised to avoid the following foods: *Solam* (Sorghum vulgare), *Kambu (*Pennisetum typhoides), *Varagu* (Paspalum scrobiculatum), *Brinjal (*Solanum melogena), *Kollu* (Macrotyloma uniflorum), *Vilangu meen* (Muraena angnilla), *Kelitru meen* (Silurus vittales), *Semmari aadu* (Ovis aries) and patient was advised to avoid prolonged standing, Frequent intake of sour and hot tastes.

## 7 Outcomes and follow-up

During this siddha intervention, patient taken these medications were completed without any complications and no ADR reported. In each visit, prognosis and symptoms are noted. In before treatment, chronic ulcer presented in right leg and swelling and hyperpigmentation presented from below the right knee joint to dorsum of right foot region associated with itching, pain and sweeling clinical features are noted. After 15 days of treatment, marginal of the ulceration is had been slightly healed and swelling and hyperpigmentation and other clinical features are mild reduced. After 60 days of treatment, ulcer healed completely and other clinical features completely recovered. In last visit, patient is advised to apply Thripala Suriya puda thylam (external use) in healed area for next 15 days. It represents Table 5.

In before treatment, DUSS score was 1. Patient had chronic non-healing ulcers in right foot region. After treatment it comes to nil. According to Wagner Ulcer Classification method before treatment patient had grade 2 ulcer (Deeper, full thickness extension). After treatment it comes to grade 0. Characteristics of Ulcer and patient satisfaction were measured by the Leg Ulcer Measurement Tool16. Clinician Rated Domains it has 14 assessment questions rated by the clinician. Clinician Rated Domains it has 14 assessment questions rated by the clinician. In before treatment Score was about 23/56 and Patient Rated Domain (PRD) it has 3 assessment questions rated by the patients. In before treatment Score was about 9/12. In before and after treatment all laboratory investigations are normal except ESR and CRP. In before treatment ESR level was 31 mm/hr and after treatment 14 mm/hr. In before treatment CRP level was 15 mm/hr and after treatment 6 mm/hr.

## 8 Discussion

The pathogenesis starts with dysfunction of venous valves causing venous hypertension which stretches the veins resulting in ulcer formation. If not treated properly, the ulcer may get infected leading to cellulitis or gangrene and eventually may need amputation of the part of limb. Venous Stasis Ulcers is called *Natipunn* [Term Id: ISMT-4.16.55] in the Siddha system of Medicine. Any ulcer developed due to pathology arising in a blood vessel and any ulcer with tortuous shape, as in a sinus or fistula is called *Natipunn. Aaraapun Natipunn* [Term Id: ISMT-4.16.50] *is described as* Chronic non-healing ulcers commonly seen in lower extremities, due to several factors affecting wound healing process [8]. In Siddha system of Medicine, three humors vali, Azal and Iyam are the basic principles of functional constituents of the body. The main cause of disease formation is the vitiation of these three humors [12]. Vali humor plays a crucial role in all physiological and biological activities of the body. Vali humor is primarily affected in Chronic ulcers. The blood vessels which contain stagnant blood become dilated, coiled and become more prominent. During this period, an inflammatory process at the site is caused by derangement of Azal. The vitiated Azal simultaneously leads to derangement of vali and Iyam which is responsible for the formation of ulcer in the affected part of the body [13].

Internal and external medicines are advised for chronic non-healing ulcers by siddha system of medicine. *Parangipattai chooranam* is a classical siddha polyherbal formulation and it indicates to *Viranam (*Ulcer), *Megam* (veneral diseases), *Venkushtam* (leucoderma) and *karunkushtam* (blackening of the skin). Promotes appetite and gives complexion to the skin. Ingredients of *Parangipattai chooranam* is *Parangipattai* (Smilax china) and *Karunthulasi* (Ocimum basilicum) [14]. It regulates the vitiated vali and Iyam humor. *Parangipattai chooranam* reported to have Anti-inflammatory, Anti-microbial, Anti- Oxidant properties help to reduce inflammations and heal ulcers. A study carried out by pushkala et al. showed that parangipattai chooranam have effective significant anti-fungal activity [15]. *Palagarai parpam* is a siddha Herbo mineral medicine and it has Anti-inflammatory, wound healing and anti-microbial activities [16]. *Palagarai* (Cypraea moneta Linn) contain 91.35% of calcium substance. Calcium ion might play a significant role in the granulation tissue development. Calcium, a free metal-containing mineral which is important for cell migration and remodelling in skin wounds [17]. Mathavan et al. study standardization of *Palagarai parpa*m showed Purification of *Palagarai* also increases its therapeutic value and if this drug is taken for the preparation of the medicine, it fulfils its therapeutic value as mentioned in the Siddha literature. *Sangu Parpam* is a Herbo mineral formulation and it indicates to treat Peptic ulcer, Skin diseases, Arthritis and Urinary Tract Infections. *Sangu parpam* has Anti-inflammatory, Antioxidant [18], Antiulcer [19] properties. *Irunelli Karpam* is a siddha Herbo mineral medicine and it has anti-oxidant property and it indicate to treats skin diseases, itching and scabies and safety drug for long time administration [20].

*Vallarai* tablet contains *Vallarai Chooranam* (*Centella asiatica*) and it indicates to treat chronic ulcers, syphilitic ulcers and lymphatic swellings [21]. *Vallarai* (centella asiatica) has antimicrobial, anti-inflammatory, anticancer, neuroprotective, antioxidant, and wound healing activities [22]. A study carried out by Afnan sh. ahmed et al. showed centella asiatica has excellent wound healing activity and its extract showed its activity in tissue regeneration, cell migration, and wound repair process by promoting fibroblast proliferation and collagen synthesis [23] and Toshiko satake et al. showed centella asiatica was shown to promote blood circulation to remove blood stasis [24].

*Thripala chooranam* has a unique poly herbal siddha formulation. It contains Kadukkai (Terminalia chebula), Nellikai (Emblica officinalis), Thantrikai (Terminalia bellirica). So many years thripala chooranam has used in external wash for ulcers. Thripala chooranam has its own antioxidant, anti-inflammatory, immunomodulating, antibacterial, antineoplastic properties. Muthusamy Senthilkumar et al. study showed triphala chooranam has good wound healing activity and reduce infections [25].

*Maththan Thylam* is a Herbo mineral Siddha formulation prescribed external application widely for several conditions such as wounds, chronic ulcers, bed sores, anal fistula, carbuncle ulcer of diabetes, per anal abscess, non-healing of external ulcers, folliculitis, and burn wound etc [26]. The ingredients of *Maththan Thylam* include coconut oil, copper sulphate, Datura metal and Acalypha indica. Histopathological study revealed, an increase in fibroblast proliferation and neo vascularization in Virgin Coconut Oil treated wounds compared to controls [27]. In the wound, Datura metal extract uses has significant inhibition on Staphylococcus Aureus, Pseudomonas Aeruginosa. Copper is an essential mineral, which plays a key role in angiogenesis, skin generation and expression and stabilization of extracellular skin proteins [28]. Acalypha indica plant extract has sufficient wound healing property [29]. A study carried out by Selvaraju et al. showed *Maththan Thylam* has an excellent rejuvenation in the wound gaping and it worked excellently in wound healing and rejuvenation [30]. Above Siddha medications are regulating the vitiated *Mukkutram* (three humors) and reduce the inflammation.

Diabetic Ulcer Severity Score (DUSS) is a standardized classification of wounds into specific subgroups of severity to compare results. Assessment using the DUSS system includes the presence of pedal pulses, the ability to probe to bone within the ulcer, and ulcer quantity and location. The sum of points determines severity, with the score ranging from 0 to 4 [31]. After treatment DUSS score comes to nil. Wagner ulcer scale is one of the oldest and most extensive categories in all types of ulcers. This scale categorizes diabetic foot ulcer on the basis of the ulcer depth into six scores: (1) grade 0: Skin is healthy; (2) grade 1: There is a superficial ulcer; (3) grade 2: There is a deep ulcer; (4) grade 3: Deep ulcer with abscess, bone involvement or osteomyelitis; (5) grade 4: Gangrene in the front or gangrene of the part of the limb (such as toe); (6) grade 5: General gangrene of the foot [32]. After treatment it comes from grade 2 to grade 0. The LUMT represents a novel assessment tool specifically designed and validated for clinical or research use on chronic leg ulcers [33]. Compared with before and after treatment, non-healing ulcer in satisfactory improvement based on assessment tools DUSS, LUMT and WUCS.

Laboratory investigations such as neutrophil-to-lymphocyte ratio, erythrocyte sedimentation rate (ESR), and C-reactive protein (CRP) level may have prognostic utility in the management of ulcerative or inflammatory conditions [34]. After treatment ESR and CRP level was significantly reduced. In ESR level reduced to14 mm/hr from 31 mm/hr and CRP level is reduced to 6 Mg/l from 15 Mg/l. It shows inflammatory conditions are reduced remarkably.

Above Siddha therapeutic regimens gave promising wound healing activities which promote venous blood circulations and reduce infections. According to this case report, after siddha intervention notable changes found in healing of venous stasis ulcer which also reduced infections. Recurrence of venous ulcer is major problem to treat or management of venous ulcers. No recurrence of ulcer was observed during the four months follow-up period of siddha intervention.

### 8.1 Conclusion

This case report strengthens the preliminary evidence to prepare the Siddha standard treatment guidelines for VLU.

One of the strengths of this case report is the patient strictly followed the prescribed diet and lifestyle modifications, and a six-month follow-up helped document the recurrence and entire procedure of the treatment. In this case, as per the patient request due to cost effective of Doppler study, previous doppler report was considered.

This case report demonstrates the application of siddha principles of diagnosis, treatments, diet and lifestyle modifications for the successful management of the chronic venous ulcers.

## 9 Patient perspective

"I had a non-healing wound, pain and itching in my right leg nearby right ankle joint. As per doctor advise, I started to take siddha medicine. After 15 days, the changes were visible, and I continued my medicine until I got recovered."

## 10 Informed consent

Written permission for publication of this case study was obtained from the patient.

## Funding sources

None.

## Data statement

Authors declare that the article submitted to the Journal of Ayurveda and Integrative Medicine is entirely our original work and we are accountable for its integrity and accuracy.

## Author contributions

**J:** Conceptualization, Methodology/Study design, Investigation, Resources, Writing – original draft, Writing – review and editing. **V:** Validation, Formal analysis, Writing – review and editing, Visualization. **S:** Supervision, Project administration.

## Declaration of generative AI in scientific writing

The authors declare that no artificial intelligence tools were used during the preparation of the manuscript.
